# *In vivo* determination of muscle-derived stem cells in the rat corpus cavernosum

**DOI:** 10.3892/etm.2014.1710

**Published:** 2014-05-14

**Authors:** LIJUN XU, BOXIN XUE, YUXI SHAN, DONG CHEN, JIE GAO, DONGRONG YANG, CHUANYANG SUN, YONG CUI

**Affiliations:** Department of Urinary Surgery, The Second Affiliated Hospital of Soochow University, Suzhou, Jiangsu 215004, P.R. China

**Keywords:** muscle-derived stem cells, rat corpus cavernosum, stem cell markers, erectile dysfunction

## Abstract

The aim of the present *in vivo* study was to determine the presence of muscle-derived stem cells (MDSCs) in the corpus cavernosum of rats. Immunohistochemistry and reverse transcription polymerase chain reaction (RT-PCR) were performed to detect the expression of the stem cell markers stem cell antigen-1 (Sca-1), Oct4 and Desmin in Sprague-Dawley rats aged 2, 5 and 20 months. Sca-1 was mainly expressed in the blood vessels and cavernous sinus and staining revealed that Sca-1 was predominantly expressed in the cytoplasm. Desmin was primarily expressed in muscular tissues and staining demonstrated that it was mainly expressed in the cytoplasm, however, Desmin was also partially expressed in the nuclei. A small number of double positive cells, expressing Sca-1 and Desmin, were also detected near the cavernous sinus. It was found that the expression of the markers was negatively correlated with the age of the rats (P<0.05). The results from the RT-PCR demonstrated that the expression levels of Sca-1 and Desmin significantly decreased with age (P<0.05). In addition, the correlation analysis indicated that the expression of Sca-1 and Desmin were negatively correlated with the age of the rats (r=−−0.929; P<0.05). In conclusion, the present study provided evidence for the presence of MDSCs in the rat corpus cavernosum. MDSCs may be a potential therapeutic treatment for organic erectile dysfunction.

## Introduction

Adult stem cells are important in the research and clinical treatment of numerous diseases (1). Adult stem cells are capable of trans-system and trans-mesoderm differentiation, and, thus, these cells have been extensively studied for a variety of diseases, including erectile dysfunction (ED) (2,3). However, the characterization and distribution of endogenous stem cells in cavernosum tissue have not been fully elucidated, and instead exogenous stem cells are utilized for investigations into cellular therapeutics for ED (4,5). These exogenous cells include embryonic stem cells, mesenchymal stem cells, adipose stem cells, muscle-derived stem cells (MDSCs) and neural crest stem cells (6). Although there are several different types of exogenous cells that may be used, the development of these cells has been limited due to the complexity and invasiveness of the treatment process (7,8). Non-invasive methods and investigations into the expression of endogenous stem cells in the cavernosum are therefore paramount for the development of successful cellular ED interventions (9).

The functional unit for erection is smooth muscle fibers and the sinusoidal endothelial cell system (10). Smooth muscle accounts for 40–50% of cavernosum tissue and is important in erectile function (11,12). Due to their ability to undergo multipotent differentiation, cavernosum MDSCs were selected as the target cells in the present study. MDSCs have been widely used in studies investigating the treatment of muscular dystrophy (8), heart disease (13), stress urinary incontinence (14), neurogenic bladder (15) as well as bone and nervous system diseases (16,17). However, despite the widespread use of MDSCs, treatments for organic ED by interfering with and regulating the expression of cavernosum MDSCs have not been reported.

The aim of the present study was to investigate the existence of cavernosum MDSCs. MDSCs are primarily obtained through short-cycle enzymatic digestion, which has the advantage of easy access and low contamination rates. The pre-plate differential adhesion technique was used for separation and purification (4,18). Since specific markers for the identification of MDSCs remain unavailable, frequently used stem cell markers, for example, stem cell antigen-1 (Sca-1), Oct4 and Desmin, were utilized (4,19,20). Immunohistochemistry and reverse transcription polymerase chain reaction (RT-PCR) were used to detect the expression of Sca-1 and Desmin in the cavernosum of rats of varying ages, as well as to investigate the distribution of MDSCs in the cavernosum tissue. The enzymatic digestion method and an improved pre-plate differential adhesion method were used to separate and purify cavernosum cells of rats. Immunofluorescence cytochemistry, flow cytometry and western blot analysis were performed to detect the expression of Sca-1, Oct4 and Desmin in adherent cells, and to further examine the associated techniques for initial separation of MDSCs. The results from the present study may provide an experimental basis for further subculture of MDSCs and induced differentiation, and provide potential therapeutic strategies for stem cell regulation therapy of organic ED.

## Materials and methods

### Animals and treatments

A total of 10 male Sprague-Dawley (SD) rats (clean grade), aged 2, 5 and 20 months were randomly selected from different nests, with average body weights of 180, 370 and 520 g, respectively. The rats were purchased from the Center of Experimental Animals at Soochow University (Suzhou, Jiangsu, China) and they were divided into young, middle-aged and old groups according to their age. The rats were subjected to anesthesia by ether inhalation and an incision was then made in the inferior portion of the abdomen. The penile tissues were carefully separated and dissected. The penis head (including the penis cartilage) and urethral sponge were removed and the corpus cavernosum was collected. Following rinsing with normal saline, the tissues were divided into two parts. The first part was immersed in 10% neutral formalin solution for fixation, hematoxylin and eosin (H&E) staining and immunohistochemical analysis. The second part was immediately placed in liquid nitrogen for storage and further analysis using RT-PCR. The current study was performed in accordance with the approved animal protocols and guidelines established by Medicine Ethics Review Committee of The Second Affiliated Hospital of Soochow University for the care and use of studied animals. All animals were given humane care in compliance with the Guide for the Care and Use of Laboratory Animals, National Institutes of Health (NIH Publication No. 85-23, revised 1996).

### Immunohistochemical analysis

Three serial sections (4 μm thick) of the cavernosum tissues were dehydrated and embedded in paraffin. The EnVision method was used for single immunohistochemical labeling of Sca-1 and Desmin, as well as for double immunohistochemical labeling of Sca-1/Desmin. Phosphate-buffered saline (PBS) was used instead of the primary antibody as the blank control.

### RT-PCR

The primer sequences were designed using Primer Premier 5.0 software (Premier Biosoft International, Palo Alto, CA, USA) and the housekeeping gene β-actin was used as an internal reference (Table I). The RT-PCR reaction system was prepared as follows: 15 μl 2X RT buffer, 1 μl primers (100 pmol/μl), 1 μl RTase, 6 μl RNA template and 7 μl diethylpyrocarbonate (DEPC)-treated water (the total volume was 30 μl). The reaction conditions were as follows: 25°C for 10 min, 40°C for 60 min and 70°C for 10 min. Preparation of the fluorescence quantitative PCR reaction system was as follows: 25 μl 2X PCR buffer, 0.6 μl primers (25 pmol/μl, ×2), 1 μl cDNA template and 22.8 μl DEPC-treated water (the total volume was 50 μl). The amplification conditions were as follows: 94°C pre-denaturation for 3 min, 94°C for 25 sec, 60°C for 25 sec and 72°C for 25 sec for 35 cycles. Agarose gel electrophoresis (2%) was used for product analysis.

### Digestion and separation of corpus cavernosum cells

Following anethesia, the penile tissue was collected under sterile conditions from the 2-month-old male SD rats. All surgical procedures were performed on an ultra-clean bench. PBS was used to rinse the tissues three times. The penis skin, subcutaneous fascia, urethra and albuginea were then carefully removed using sterile ophthalmic scissors, and the corpus cavernosum tissues were stored. The cavernous tissues were cut into small 1–2 mm^3^ sections and enzymes were used to digest and separate the cells. The tissue sections were first digested with type I collagenase (0.5%) at a constant temperature for 3 h. Trypsin (0.1%) was then added in the same volume and the tissues were digested for a further 30 min. The cells were then observed under a microscope (Axiovert 100, Zeiss, Oberkochen, Germany) and high glucose Dulbecco’s modified Eagle’s medium (DMEM) containing 10% fetal calf serum (FCS; volume fraction; volume fraction was identical to volume concentration in ideal solutions, where the constituents volumes are additive that is equal to the total volumes of its ingredients) was added to terminate the digestion when nearly all the cells were dispersed into single cells. The solution was then filtered using a 200-mesh filter and 200 g of filtrate was collected and centrifuged at 200 × g for 10 min. The supernatant was discarded and the cell pellet was resuspended in DMEM containing 20% FCS (volume fraction). The cell concentration was adjusted and inoculated in the cell culture bottles and the cells were incubated at 37°C and 5% CO_2_ (volume fraction).

### Purification and culture of cells

The contaminated cells were removed using the pre-plate differential adhesion method (21). The cells were cultured for 1 h and the adherent cells were considered pre-plate 1 (PP1). The non-adherent cells were cultured in a new culture bottle for a further 2 h and the adherent cells were considered pre-plate 2 (PP2). The non-adherent cell suspension was transferred into a new bottle and cultured for a further 18 h, and the subsequent adherent cells were considered pre-plate 3 (PP3). Following this, transfer bottle cultures were then performed every 24 h and the cells were successively identified as pre-plate 4, 5 and 6, respectively (PP4, PP5 and PP6). PP6 cells began to adhere to the wall following 2–3 days and the culture solution was changed once a day. The cells were observed under an inverted microscope and divided and cultured in different bottles when the cell confluence approached 50%. These cells were then used for the subsequent experiments.

### Flow cytometric analysis

PP6 cells were collected and the cell concentration was adjusted to 1×10^10^/l. Reagent A (100 μl) was added for fixation for 15 min and the cells were rinsed once with PBS. The cells were subsequently centrifuged and collected and the primary antibodies (Sca-1, Oct4 and Desmin; 1:50) were added. The immunoglobulin G (IgG) corresponding to the primary antibody was added to the control group and fully mixed with the cells. The cells were incubated at room temperature for 40 min and then washed twice with PBS prior to the addition of fluorescein isothiocyanate (FITC)-labeled secondary antibody corresponding to the primary antibody (1:50). The cells were incubated at room temperature in the dark for 30 min. Subsequently, the cells were washed once with PBS and 500 μl PBS was added. A flow cytometer was then used to detect the positive cell count.

### Immunofluorescence cytochemical identification

The PP6 cells were inoculated in a six-well plate with a cover glass. After the cells adhered to the cover glass, the growth cover glasses were prepared. A total of 40 g/l paraformaldehyde was used to fix the cells for 2 h and the cells were then immersed and rinsed with PBS. Triton X-100 (1%) was added and the cells were incubated at room temperature for 15 min. The cells were then immersed and rinsed again with PBS. The growth cover glasses were treated with 3% H_2_O_2_-methanol solution (volume fraction) for 15 min following which the cells were then immersed and rinsed with PBS. A total of 100 μl primary antibody was added (Sca-1, embryonic antigen and Desmin; 1:50) and the cells were incubated at 37°C for 2 h. The two types of primary antibodies were added simultaneously at the same time as Sca-1/Oct4, Sca-1/Desmin and Oct4/Desmin double immunofluorescence cytochemical analysis. PBS was used instead of the primary antibody as the negative control and the cells were immersed and rinsed with PBS. Then, 100 μl FITC-labeled secondary antibody (1:200) corresponding to the primary antibody was added and the cells were incubated at 37°C for 1 h. The two types of secondary antibodies, corresponding to the primary antibody, were added simultaneously at the same time as the double-label immunofluorescence method. PBS was used to immerse and rinse the cells and the mounting medium preventing fluorescence quenching was used. The specimens were then observed and images were captured under a fluorescence microscope (Carl Zeiss MicroImaging Inc., Thornwood, NY, USA). The images were processed using Image-Pro Plus software (Media Cybernetics, Silver Spring, MD, USA).

### Western blot analysis

PP1–PP6 cells were collected and a total protein extraction kit was used to extract the proteins in the different cell samples. The Bradford method was used to determine the protein concentrations of different samples and the loading amount was adjusted. Electrophoresis was performed using an SDS-PAGE gel. The proteins were transferred onto a PVDF membrane and inhibited using the blocking buffer. Following this, the primary antibody (Sca-1, Oct4 and Desmin; 1:400) was added and the membranes were incubated on a shaker at 4°C overnight. The secondary antibody [horseradish peroxidase (HRP)-goat anti-rabbit IgG and HRP-sheep anti-mouse IgG; 1:5,000 dilution with blocking buffer] was then added after the membrane was rinsed and exposure was performed which was developed.

### Determination of positive results

The immunohistochemical results were collected from the different groups and analyzed under a microscope (magnification, ×400; JEOL, Model JSM-7600F). The positive staining result for single labeling was brown and the positive staining result for double labeling was dark brown.

### Statistical analysis

The data are presented as the mean ± standard deviation. T-tests were used to determine differences between groups and P<0.05 was considered to indicate a statistically significant difference. Spearman rank correlation was used for the correlation analysis and P<0.05 was considered to indicate a statistically significant difference.

## Results

### H&E staining

**Figure 1:**
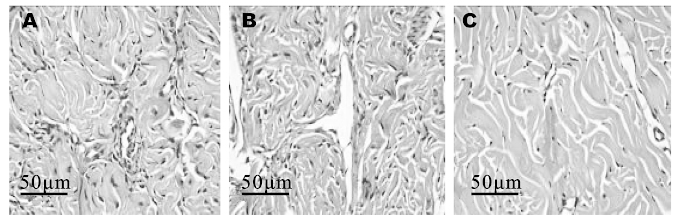
Differences in structural density of the corpus cavernosum and the distribution of blood vessels in Sprague-Dawley rats aged (A) two (young group), (B) five (middle-aged group) and (C) 20 months (old group). The cells were stained with hematoxylin and eosin (magnification, ×400).

The corpus cavernosum of the rats gradually changed from tightly organized structures to loosely organized structures as the age of the rats increased. Similarly, the blood vessels were found to be abundant, slightly decreased and significantly decreased in the young, middle-aged and old rats, respectively (Fig. 1).

### Immunohistochemical results

**Figure 2:**
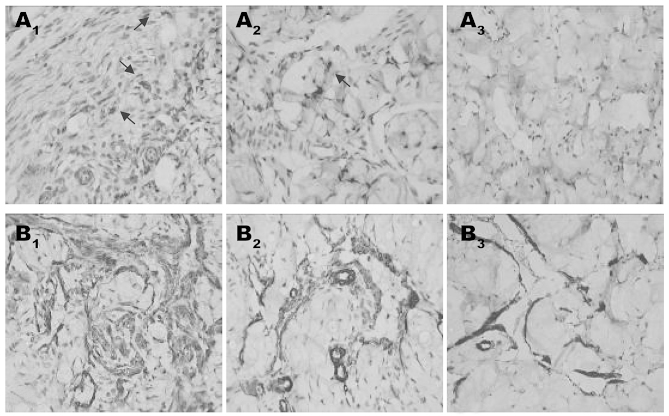
Expression of (A) Sca-1 and (B) Desmin in the corpus cavernosum of Sprague-Dawley rats of different ages (magnification, ×400). 1, 2 and 3 correspond to the young, middle-aged and the old groups, respectively. The arrows indicate positively stained cells. Sca-1, stem cell antigen-1.

**Figure 3:**
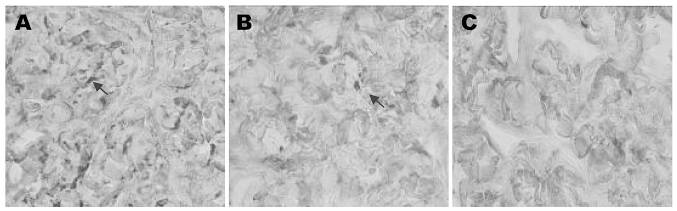
Expression of Sca-1/Desmin in the corpus cavernosum of Sprague-Dawley rats aged (A) two (young group), (B) five (middle-aged group) and (C) 20 months (old group). The arrows indicate positively stained cells (magnification, ×400). Sca-1, stem cell antigen-1.

The immunohistochemical results demonstrated that Sca-1 was predominantly expressed in the blood vessels and cavernous sinus, and demonstrated primarily cytoplasmic staining. By contrast, Desmin was expressed primarily in muscular tissues and staining demonstrated that Desmin was mainly expressed in the cytoplasm, however, it was also partially expressed in the nuclei. A small number of double positively stained cells (Sca-1/Desmin) were also detected near the cavernous sinus. A statistically significant difference in the expression of Sca-1 and Desmin between the different age groups was also observed (P<0.05). Expression of the markers was found to be negatively correlated with the age of the rats (P<0.05; Fig. 2 and 3).

### RT-PCR results

The results from the RT-PCR demonstrated that the expression levels of Sca-1 and Desmin significantly decreased with age (P<0.05). Significant differences in the concentrations of the markers were identified between the different age groups (P<0.05; Table II), which was in accordance with the results from the immunohistochemical analysis.

### Correlation analysis

The results from the correlation analysis indicated that the expression of Sca-1 was significantly and negatively correlated with the age of the rats (r=−0.929; P<0.05). Similarly, the same result was observed for Desmin (r=−0.924; P<0.05).

### Cell isolation and flow cytometry

**Figure 4:**
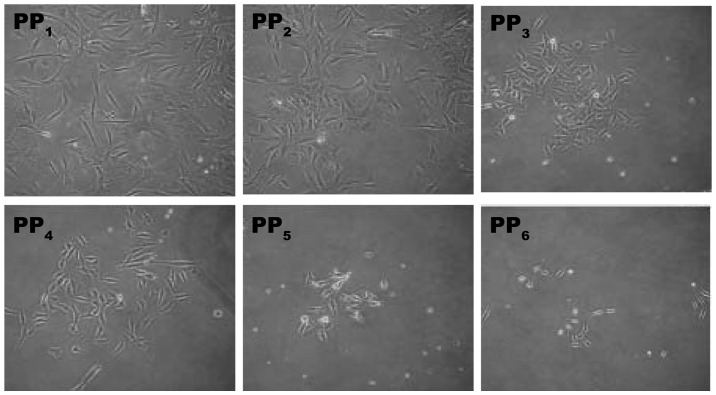
Morphology of PP1–PP6 cells under the light microscope (magnification, ×400). PP, pre-plate.

PP1 and PP2 cells adhered to the wall rapidly and they were primarily long spindle-shaped fibrous cells. The adherent capability of PP3–PP5 cells successively decreased and in these plates, short spindle-shaped cells were observed. Several of these cells were polygon-shaped and primarily consisted of vascular endothelial and smooth muscle cells. A number of the PP6 cells were small, round floating cells. These PP6 cells slowly adhered to the wall and became round or spindle-shaped following 2–3 days (Fig. 4).

### Immunofluorescence cytochemistry

**Figure 5:**
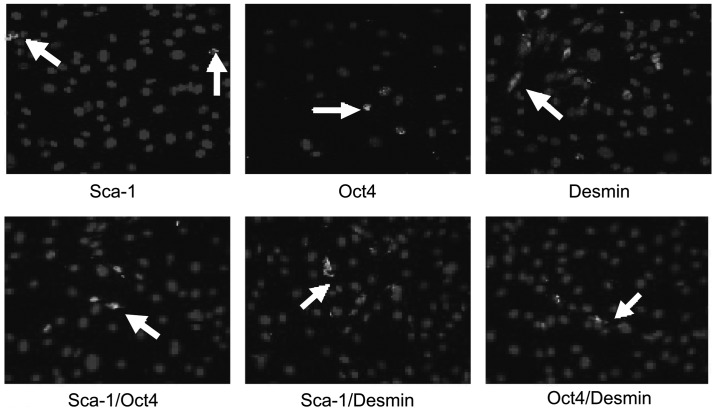
Expression of Sca-1, Oct4, Desmin, Sca-1/Oct4, Sca-1/Desmin and Oct4/Desmin in the pre-plate 6 cells. The arrows indicate positive cells (magnification, ×400). Sca-1, stem cell antigen-1.

**Figure 6:**
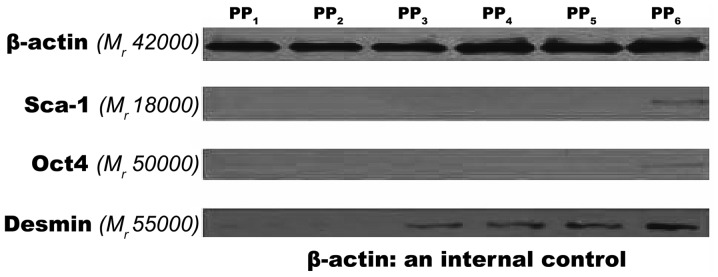
Western blot analysis of Sca-1, Oct4 and Desmin expression in PP1–PP6 cells. PP, pre-plate; Sca-1, stem cell antigen-1.

The expression of Sca-1, Oct4 and Desmin was detected in PP6 cells, however, the expression of Sca-1 and Oct4 was only detected in a few cells. The expression of Sca-1, Oct4 and Desmin in PP6 cells was 5.7, 2.6 and 41.2% respectively (Fig. 5). Sca-1 and Desmin were primarily expressed in the cytoplasm, whilst Oct4 was primarily expressed in the nuclei (Fig. 6). A small number of cells expressed Sca-1/Oct4, Sca-1/Desmin and Oct4/Desmin. Sca-1/Oct4 were expressed in the cytoplasm and the nuclei, whilst Sca-1/Desmin were primarily expressed in the cytoplasm. Oct4/Desmin were mainly expressed in the nuclei and the cytoplasm.

### Western blot analysis

When the total amount of the proteins was equivalent, significant expression levels of Sca-1 and Oct4 were not detected in the PP1–PP5 cells. However, the expression of Sca-1 and Oct4 was detected in the PP6 cells and the concentrations were lower than the internal reference. A very low expression level of Desmin was detected in PP1–PP2 cells, while its expression level significantly increased in PP3–PP6 cells (Fig. 6).

## Discussion

The present study investigated the expression and distribution of Sca-1, Desmin and Sca-1/Desmin in the corpus cavernosum of rats. It was found that the expression levels were significantly negatively correlated with the age of the rats, suggesting that the expression of these markers gradually decreases with age. As part of this investigation, the corpus cavernosum cells were also isolated and purified to determine the expression of Sca-1, Oct4, Desmin, Sca-1/Oct4, Sca-1/Desmin and Oct4/Desmin. These data provide evidence for further subculture and amplification of MDSCs, and also provide evidence for possible therapeutic strategies for non-invasive stem cell regulatory therapy for organic ED.

In the present study, important techniques were used to isolate MDSCs in corpus cavernosum tissue. The corpus cavernosum tissues were processed using enzymatic digestion. The modified corpus pre-plate differential adhesion technique was further utilized to isolate and purify the MDSCs as described previously by Zuba-Surma *et al* (22). This technique provides a homogenous sample of stem cells (23). Fibroblasts and collagen fiber cells have the greatest adhesive capability; therefore, these cells were primarily isolated on the plate. Since the adhesive capability of vascular endothelial cells and smooth muscle cells is relatively low, these cells adhere on later plates. MDSCs have the lowest adhesion capability, therefore, following continuous six-step differential adhesion, the majority of impure cells were removed. Detection efficiency can be improved in the final sample of cells. The isolated PP6 cells in the present study were small and round or short fusiform-shaped cells and their adhesive capability was relatively weak, which was consistent with the characteristics of MDSCs.

In the present study, the expression of Sca-1 and Oct4 was detected in cavernous tissue and cells, and the results obtained were consistent with previous studies by Zuba-Surma *et al* and Ho *et al* (22,24) investigating non-corpus cavernosum-derived MDSCs. The techniques presented in the present study may be used with previously established methods to identify corpus cavernosum stem cells and improve the identification rate of these stem cells.

Previous studies have indicated that that the positive rate of Desmin in MDSCs can be as high as 90%. Therefore, Desmin is frequently used for the identification of muscle-derived MDSCs (25). In the present study, very few double positive cells (Sca-1/Desmin) were detected near the cavernous sinus. The double positive cells (Sca-1/Oct4, Sca-1/Desmin and Oct4/Desmin) were successfully isolated, which further confirmed that MDSCs existed in the stem cells from corpus cavernosum. However, it is important to note that endothelial, vascular, neurological and other factors are also important in the pathogenesis of organic ED (26). Whilst MDSCs possess the multiple differentiation capability of stem cells, they not only differentiate into myogenic cells, but also have the potential to differentiate into endothelial, vascular and neural cells. Therefore, these cells may be important in future clinical treatment approaches.

Another important finding from the present study was the association between the expression of the markers and the age of the rats. The expression of the markers was significantly decreased in the old group compared with the young group of rats. This suggests that the collection of stem cells from the corpus cavernosum of rats should be performed on young rats. In addition, the efficacy of regulatory therapy of ED using endogenous stem cells may be closely associated with the age of patients. The therapeutic efficacy may be higher in middle-aged and younger patients compared with older patients.

The present study provides an important foundation for future studies that target cellular treatments for ED. In the present study, corpus cavernosum MDSCs were detected and isolated on the tissue level, which is a promising first step for future treatments for ED using endogenous stem cells. However, the subculture amplification, multiple-direction induced differentiation and functional tests on animal models require further investigation before conclusions can be made regarding the viability MDSCs. The incidence of diabetic-associated ED has increased in recent years and it has been attributed to structural impairments in endothelial cells, decreased smooth muscle cells and cavernous nerve injury (27,28). However, the potent self-renewing and proliferative capability of MDSCs, as well as the multi-direction differentiation potency, may have the potential to treat diabetic ED. Certain studies have proposed that endogenous organ stem cells may be activated by ultrasound treatments on the distribution region of stem cells (29). This may provide a feasible treatment paradigm for the non-invasive regulatory therapy of organic ED using stem cells in the future.

## Figures and Tables

**Table I:** Primer sequences for the detection of mRNA expression level and product length.

Gene	Sense	Anti-sense	Product length (bp)
β-actin	CCCATCTATGAGGGTTACGC	TTTAATGTCACGCACGATTTC	150
Sca-1	AACCATATTTGCCTTCCCGTCT	CCAGGTGCTGCCTCCAGTG	135
Desmin	CTTGATGAGGCAGATGAGGA	AGCTTCCGGTAGGTGGCAAT	192

Sca-1, stem cell antigen-1.

**Table II:** Expression levels of Sca-1 and Desmin in the corpus cavernosum of rats from different age groups.

Group	Young group	Middle-aged group	Old group
Sca-1	0.55±0.07	0.27±0.04^a^	0.14±0.02^a^,^b^
Desmin	3.40±0.31	2.10±0.23^a^	1.10±0.24^a^,^b^

a P<0.05, compared with the young group;

b P<0.05, compared with the middle-aged group.
